# Author Correction: The multi-mode acoustic gravitational wave experiment: MAGE

**DOI:** 10.1038/s41598-024-55260-w

**Published:** 2024-02-27

**Authors:** William M. Campbell, Maxim Goryachev, Michael E. Tobar

**Affiliations:** ARC Centre of Excellence for Engineered Quantum Systems and ARC Centre of Excellence for Dark Matter Particle Physics, Department of Physics, University of Western Australia, 35 Stirling Highway, Crawley, WA 6009 Australia

Correction to:* Scientific Reports* 10.1038/s41598-023-35670-y, published online 30 June 2023

The original version of this Article contained errors.

Equation 4 was incorrectly given as:

$$\begin{array}{c}{S}_{h}^{+}\left(\omega \right){|}_{\omega ={\omega }_{\lambda }}=\sqrt{\frac{{k}_{b}{T}_{\lambda }{\omega }_{\lambda }}{{Q}_{\lambda }{M}_{\lambda }}}\left(\frac{1}{{\omega }_{\lambda }^{2}{h}_{0}\xi }\right).\end{array}$$

The correct Equation 4 appears below.

$${S}_{h}^{+}\left(\omega \right){|}_{\omega ={\omega }_{\lambda }}=\sqrt{\frac{4{k}_{b}{T}_{\lambda }{\omega }_{\lambda }}{{Q}_{\lambda }{M}_{\lambda }}}\left(\frac{1}{{\omega }_{\lambda }^{2}{h}_{0}\xi }\right).$$

In addition, in the Sensitivity to HFGWs section,

“Based on the discussions presented in Aggarwal *et al*^8^, and using the C_3,0,0_ mode as an example, MAGE would be sensitive to a 0.004 ${M}_{\cdot }$ black hole merger, where ${M}_{\cdot }$ denotes the solar mass.”

now reads:

“Based on the discussions presented in Aggarwal *et al*^8^, and using the C_3,0,0_ mode as an example, MAGE would be sensitive to a 0.0004 ${M}_{\odot }$, where ${M}_{\odot }$ is the solar mass.”

The original Article has been corrected.

